# Early endoscopic management for early bowel obstruction after gastrectomy: a case report

**DOI:** 10.1186/s40792-016-0164-3

**Published:** 2016-04-12

**Authors:** K. Higashizono, S. Aikou, K. Yagi, K. Mori, H. Yamashita, S. Nomura, Y. Seto

**Affiliations:** Department of Gastrointestinal Surgery, The University of Tokyo Hospital, 7-3-1 Hongo, Bunkyo-ku, Tokyo, 113-8655 Japan

**Keywords:** Gastrectomy, Early bowel obstruction, Endoscopy

## Abstract

**Background:**

Early bowel obstruction is not a rare complication of gastrectomy, and it may require re-operation in some cases.

**Case presentation:**

We report the case of a 71-year-old woman who underwent a total gastrectomy with Roux-en-Y reconstruction for a massive gastrointestinal stromal tumor. Postoperatively, she was making good progress and started consuming meals on postoperative day 3. However, on postoperative day 10, she complained of upper abdominal discomfort and nausea. Blood tests showed a mild inflammatory reaction. An upper gastrointestinal series showed obstruction of the elevated jejunum. An abdominal computed tomography scan suggested upper bowel obstruction. Endoscopic observation and repositioning was selected as an effective approach for treatment considering the patient’s clinical condition and background. Upper gastrointestinal endoscopy showed kinking of the elevated jejunum, easy passage through to the anal intestine, and no evidence of mucosal edema, stenosis of the Roux-en-Y anastomosis, bowel ischemia, or necrosis. After endoscopic repositioning, upper gastrointestinal series showed good passage of the jejunum and no evidence of bowel obstruction. At the 6-month follow-up examination, the patient was in good condition and had no complaints.

**Conclusion:**

We concluded that early endoscopic management should be the effective procedure considered for diagnosis and treatment of early bowel obstruction after gastrectomy in some cases.

## Background

Gastrectomy is associated with several postoperative complications, and early gastrointestinal obstruction is not a rare complication and generally occurs 2 weeks after surgery [1–3]. Most patients diagnosed with early bowel obstruction after gastrectomy are observed conservatively and do not require surgical intervention. However, some need re-operation for both diagnosis and treatment when their situation does not improve [4]. Here, we present a case of early gastrointestinal obstruction after gastrectomy with Roux-en-Y reconstruction that was diagnosed and treated early with upper gastrointestinal endoscopic repositioning. After this intervention for the complication, the patient had a good clinical course.

## Case presentation

**Fig. 1:**
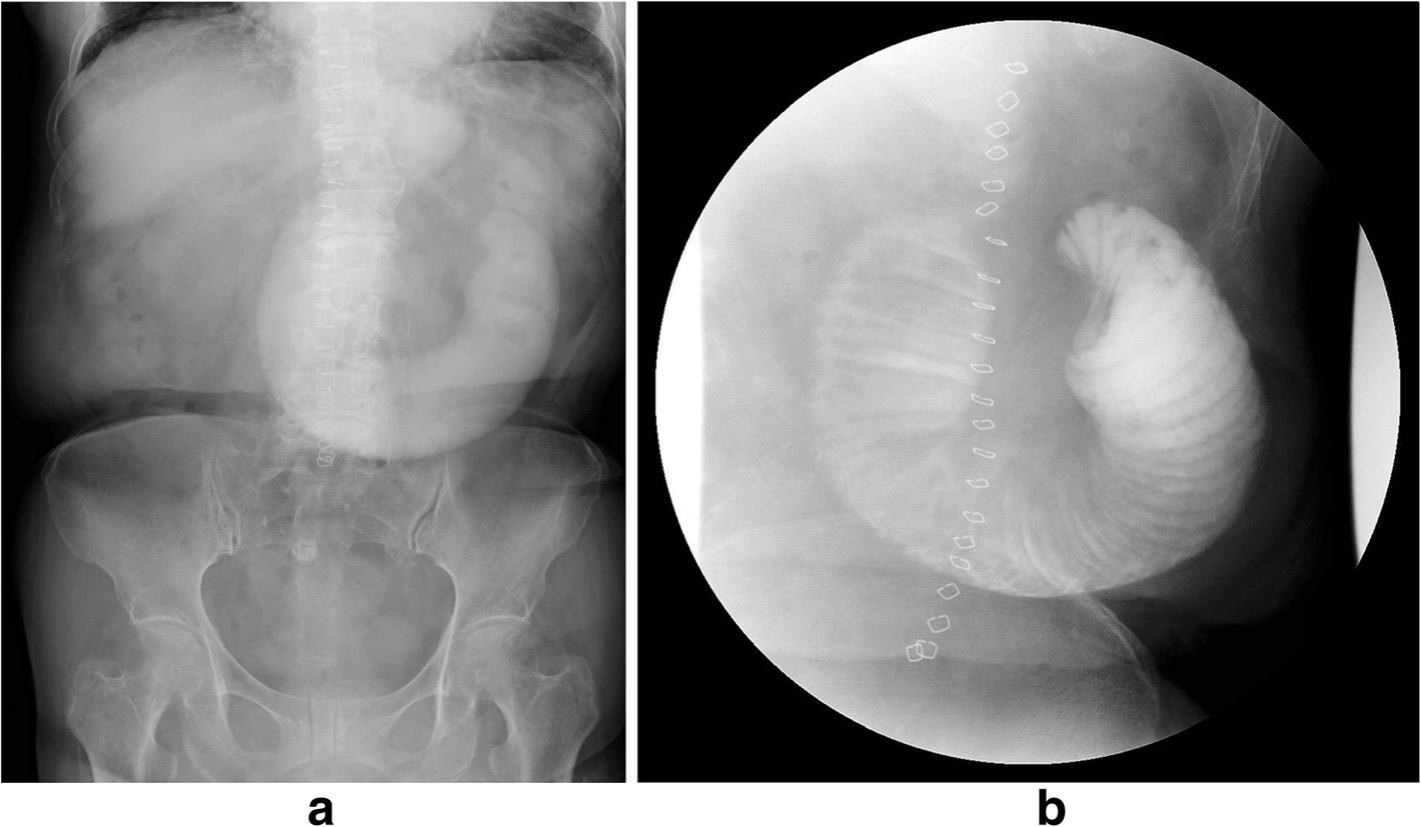
Abdominal X-ray picture and upper gastrointestinal series findings. Abdominal X-ray picture (**a**) and upper gastrointestinal series (**b**) showed decreased peristalsis and distension of the elevated jejunum without visible passage to the anal intestine

**Fig. 2:**
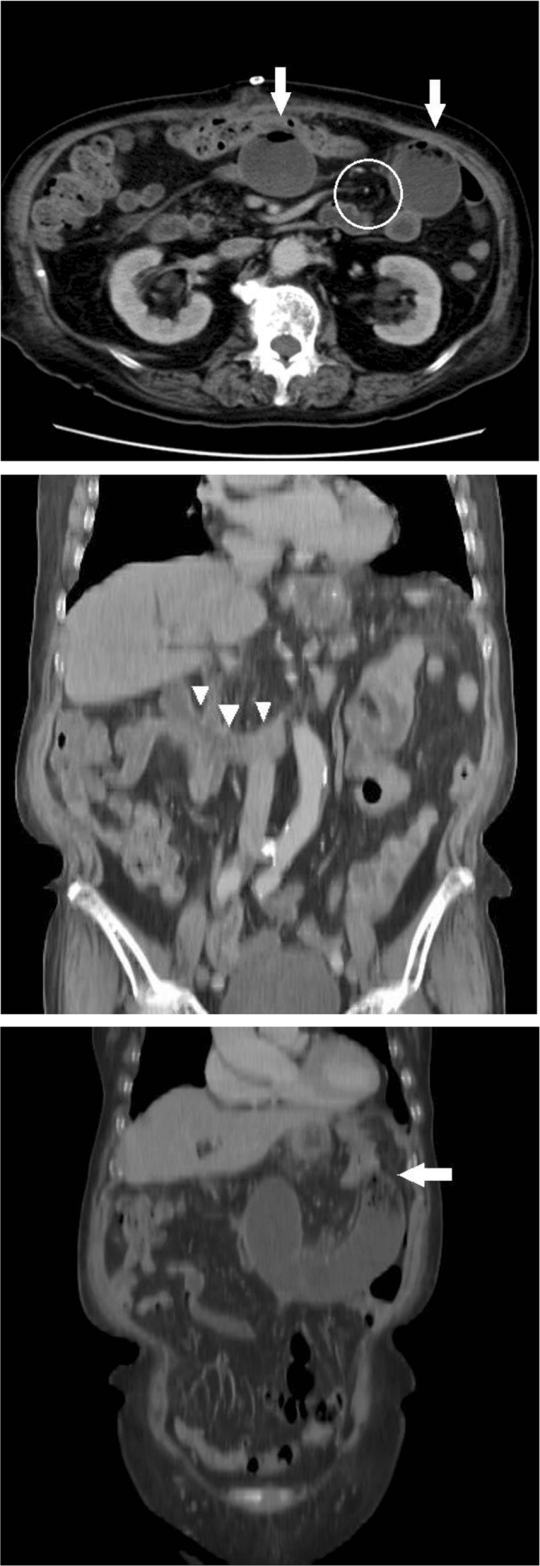
CT scan findings. Axial CT scan shows crowding of the main mesenteric trunk to the left and distention of the elevated jejunum (*white arrow*). A swirled appearance of the mesenteric fat and vessels was found (*white circle*). Coronal CT scan shows no distension of the jejunum around the Treitz ligament (*white arrowheads*). The obstruction was located at the elevated jejunum about 10 cm from the Y anastomosis. *White arrow* shows the kinking point of elevated jejunum in coronal CT scan

**Fig. 3:**
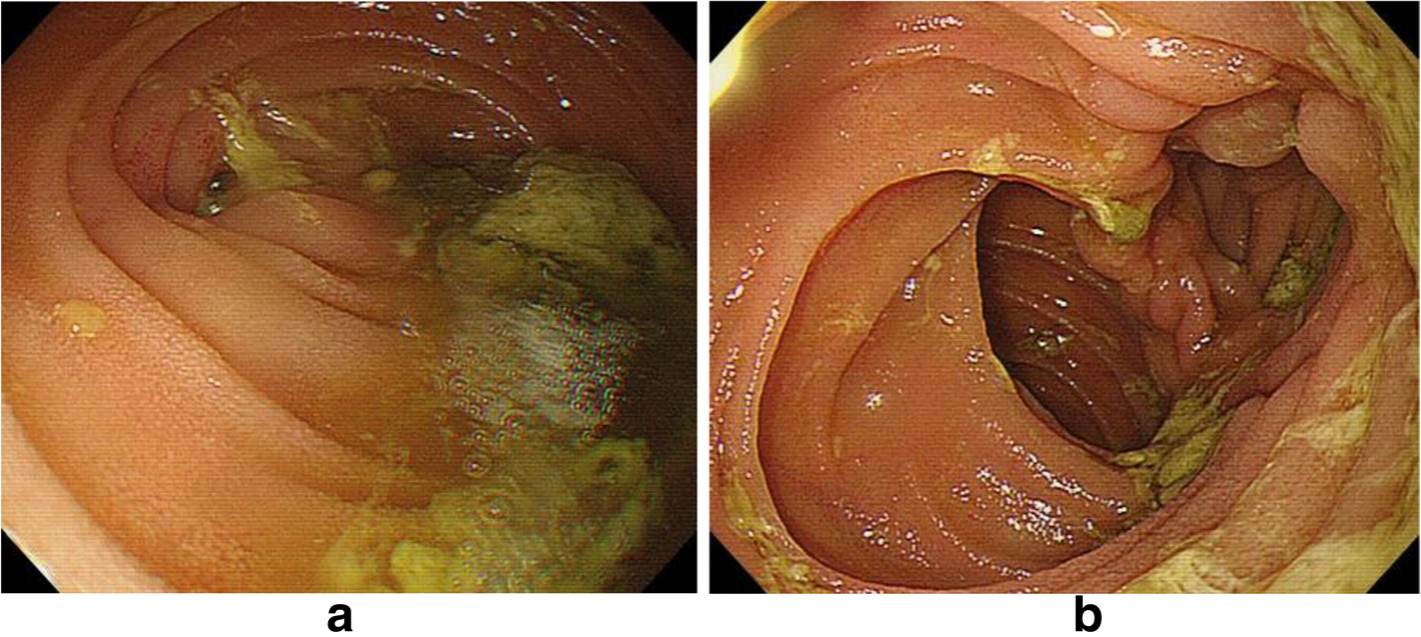
Endoscopic findings. Endoscopic findings showed slight kinking of the elevated jejunum (**a**), and the endoscope easily passed through to the anal intestine and no evidence of mucosal edema or stenosis of Roux-en-Y anastomosis, bowel ischemia, or necrosis (**b**)

**Fig. 4:**
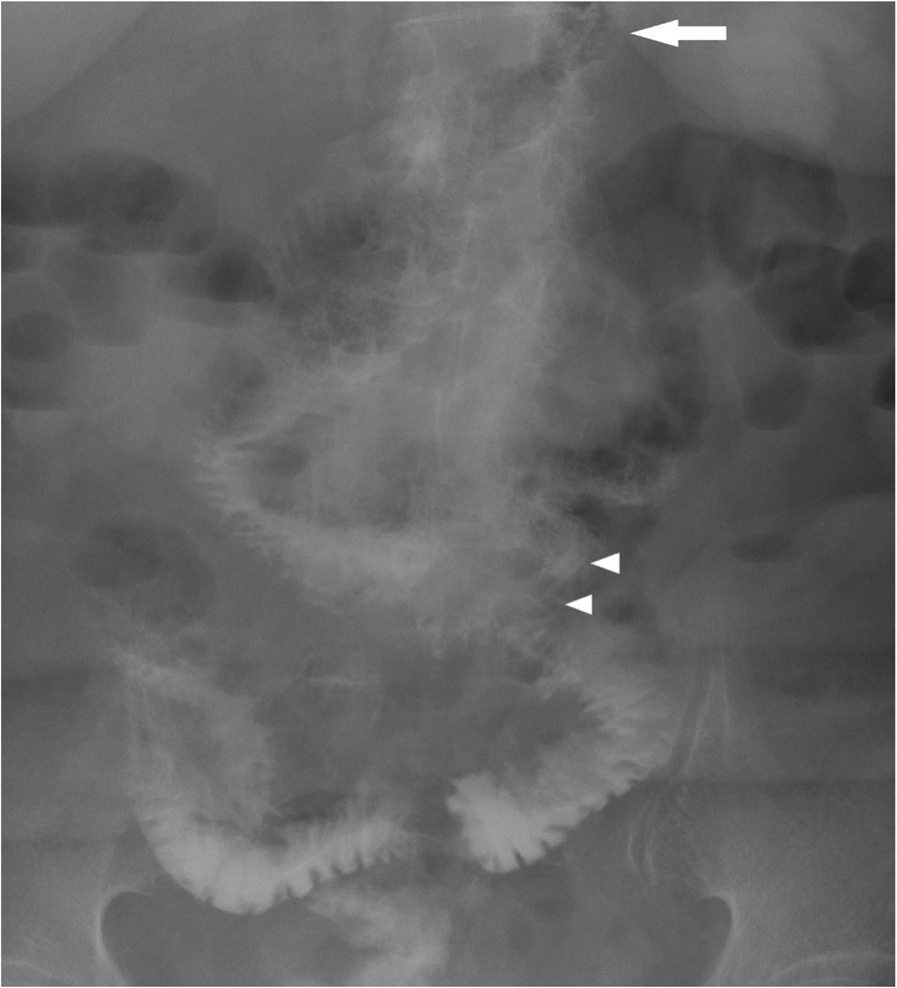
Upper gastrointestinal series after endoscopic repositioning. After endoscopic repositioning, the elevated jejunum shifted to the left, and there was good passage to the jejunum and no evidence of bowel obstruction (*white arrow*: esophagojejunostomy, *white arrowheads*: jejunojejunostomy)

A 71-year-old woman was referred to our hospital to undergo surgery for a gastrointestinal stromal tumor (GIST; intermediate risk group, positive for c-Kit and CD34 and negative for S100 and 1A4 with nuclear segmentation <5/50 high-power field). She suffered from deep vein thrombosis (DVT) after undergoing total hip arthroplasty 6 months previously and was receiving oral anticoagulant therapy. We performed total gastrectomy because it was the massive GIST, which was 9.0 × 9.5 × 5.0 cm in size and was located in the anterior side of the stomach and 2 cm from the gastroesophageal junction. Roux-en-Y reconstruction with the retrocolic route was conducted. The jejunojejunostomy was located 40 cm from esophagojejunostomy, and petersen defect and jejunojejunal mesenteric defect were closed during the surgical procedure. The patient was making good progress after the operation and started consuming meals on postoperative day 3. However, on postoperative day 10, she complained of upper abdominal discomfort and nausea. Physical examination showed partial abdominal tenderness and no rebound tenderness, with no rigidity, and the patient had a fever (38.2 °C). The white blood cell count was 9100/μL (normal range, 4000–8000/μL), and the C-reactive protein level was 3.0 mg/L (normal range, 0–0.5 mg/L), indicating mild inflammation; no remarkable abnormalities were found in other biochemical tests including renal, hepatic function, and blood gas tests. An upper gastrointestinal series (UGIS) and abdominal X-ray picture showed decreased peristalsis and distension of the jejunum with no visible passage to the anal intestine (Fig. 1a, b). Computed tomography (CT) scans showed signs of small bowel obstruction without strangulation. There was no distension of the jejunum around the Treitz ligament. The obstruction was located at the elevated jejunum about 10 cm above the Y anastomosis. On postoperative day 10, she underwent nasogastric decompression and bowel rest but her situation did not improve. Because the patient was elderly and at a high risk for re-operation under anticoagulant therapy, and no evidence of bowel strangulation or necrosis was noted (Fig. 2), endoscopic observation and repositioning were conducted on postoperative day 13. Endoscopic findings showed slight kinking of the elevated jejunum 70 cm from the mouth (Fig. 3a). We performed endoscopic repositioning, pushed slowly with less air, twisted the shaft to the right without radiographic guidance, and obtained easy passage through to the anal intestine with no evidence of mucosal edema, stenosis of the Roux-en-Y anastomosis, bowel ischemia, or necrosis (Fig. 3b). The esophagojejunostomy was located 40 cm from the mouth, and the jejunojejunostomy was located 80 cm from the mouth. On the day following the endoscopic repositioning, the UGIS showed good passage from the esophagus to the jejunum and no evidence of bowel obstruction (Fig. 4). The patient began to eat 1 day after endoscopic repositioning, the abdominal discomfort did not recur, and she was discharged on postoperative day 17. She was followed up for 6 months and experienced no abdominal pain episodes or other complications.

## Conclusions

Recent studies have reported that endoscopic repositioning should be the first procedure considered for patients with lower gastrointestinal conditions like sigmoid colon volvulus [5]. However, few studies have reported endoscopic repositioning for early bowel obstruction after gastrectomy. In the present case, we safely conducted endoscopic repositioning for this condition, and the patient recovered rapidly.

Bowel obstruction after gastrectomy can be an early or late complication, with the 2-week period after surgery as the threshold [2, 6, 7]. Late bowel obstruction is treated using endoscopic ballooning or stenting when mechanical stenosis is found [10]. Early bowel obstruction is not a rare and can be caused by technical problems, such as bowel kinking, postsurgical anastomotic edema, stenosis, ischemia, and rarely, internal herniation. In many cases, nasogastric decompression and bowel rest may assist in resolution [7], but when the patient’s condition does not improve, re-operation is required. Several studies have indicated that surgical exploration should be performed as early as possible in patients with early bowel obstruction, especially those suspected of having an internal hernia with ischemia [8, 9].

In the present case, we could not ascertain the cause of bowel obstruction, although imaging findings suggested the possibility of internal herniation or bowel obstruction due to banding or adhesion of the elevated jejunum. We closed possible hernia sites during the surgical procedure to prevent internal herniation. Therefore, we could not arrive at a conclusion whether possible hernia sites should be closed during operation. In our cases, we should have shortened the elevated jejunum in surgical procedure.

There was no evidence of strangulation, bowel ischemia, or perforation in the images. Nasogastric decompression and bowel rest did not improve her situation. Her condition was stable with no abdominal tenderness, and further, she had several surgical risk factors and needed to avoid re-operation. We selected endoscopy for observation and treatment because it has several merits: endoscopy ensures (1) viability of the bowel, shows the exact location of the damage and provides information regarding the mucosal appearance; (2) allows mechanical release of bowel obstruction and decompression of the dilated proximal bowel; and (3) is less invasive and more convenient than surgery [10]. In fact, in the present case, the endoscopic findings showed slight kinking of the elevated jejunum, easy passage through to the anal intestine, and no evidence of bowel ischemia or necrosis. The patient was relieved of her symptoms after endoscopic repositioning and recovered by avoiding operation. Of course, we think endoscopic management should not be performed when the patient is at high risk of aspiration or there is the possibility of bowel ischemia or necrosis. When repositioning did not succeed, we have to consider surgical intervention. In addition, if gastrointestinal necrosis or bowel strangulation is strongly suspected on CT scans and/or from clinical manifestations, immediate surgical intervention is required. Our patient is currently under observation, but she has been followed up for 6 months and has not shown recurrence of bowel obstruction. From this experience, we concluded that early endoscopic management should be the first choice for diagnosis and treatment of early bowel obstruction after gastrectomy.

## Consent

Written informed consent was obtained from the patient for publication of this case report and any accompanying images. A copy of the written consent is available for review by the Editor of this journal.

## Abbreviations

CT: computed tomography
DVT: deep vein thrombosis
GIST: gastrointestinal stromal tumor
UGIS: upper gastrointestinal series
